# Limb proportions show developmental plasticity in response to embryo movement

**DOI:** 10.1038/srep41926

**Published:** 2017-02-06

**Authors:** A. S. Pollard, B. G. Charlton, J. R. Hutchinson, T. Gustafsson, I. M. McGonnell, J. A. Timmons, A. A. Pitsillides

**Affiliations:** 1 Comparative Biomedical Sciences, the Royal Veterinary College, London, NW1 0TU, UK; 2 Genetics and Molecular Medicine, King’s College London, London, WC2R 2LS, UK; 3 Department of Laboratory Medicine, Karolinska University Hospital, 14186, Stockholm, Sweden

## Abstract

Animals have evolved limb proportions adapted to different environments, but it is not yet clear to what extent these proportions are directly influenced by the environment during prenatal development. The developing skeleton experiences mechanical loading resulting from embryo movement. We tested the hypothesis that environmentally-induced changes in prenatal movement influence embryonic limb growth to alter proportions. We show that incubation temperature influences motility and limb bone growth in West African Dwarf crocodiles, producing altered limb proportions which may, influence post-hatching performance. Pharmacological immobilisation of embryonic chickens revealed that altered motility, independent of temperature, may underpin this growth regulation. Use of the chick also allowed us to merge histological, immunochemical and cell proliferation labelling studies to evaluate changes in growth plate organisation, and unbiased array profiling to identify specific cellular and transcriptional targets of embryo movement. This disclosed that movement alters limb proportions and regulates chondrocyte proliferation in only specific growth plates. This selective targeting is related to intrinsic mTOR (mechanistic target of rapamycin) pathway activity in individual growth plates. Our findings provide new insights into how environmental factors can be integrated to influence cellular activity in growing bones and ultimately gross limb morphology, to generate phenotypic variation during prenatal development.

A huge diversity of limb specializations exists in nature. All tetrapods (vertebrates with limbs having digits, ancestrally) possess an equivalent basic limb design, comprising skeletal elements that originate during embryonic development from the three major limb regions (the stylopod, zeugopod and autopod)^1^. These elements are tailored in their proportions to fulfil a range of roles^2^,^3^,^4^, first emerge during prenatal development to characterise each species^5^,^6^, and also vary to some extent within species^7^,^8^,^9^,^10^. It is well established that natural selection has led to limb proportions that are adapted to improve locomotor performance in specific environments on an evolutionary time-scale^11^,^12^,^13^,^14^. However, the extent to which these limb proportions are also influenced by shorter-term alterations in the environment within the lifetime of a single organism has historically not been as well resolved.

Dramatic evidence has recently emerged which indicates that environmental cues engender profound changes in behaviour and musculoskeletal form post-natally. These changes are analogous to adaptive changes inferred in evolution and are seen, for example, in the modification of bone shape and limb function necessary for the terrestrialization of *Polypterus* fish^15^. Morphological changes including alterations in limb/extremity proportions have also been observed in mammals raised in different climates^16^,^17^. We speculate that this phenotypic plasticity during prenatal periods of ontogeny could also confer an ability to incorporate shorter-term environmental input into the generation of variation even prior to birth/hatching. Whether such interactions between environmental input and ‘intrinsic’ aspects of developmental regulation facilitate phenotypic variation in limb morphology during prenatal development has not however previously been explored.

Morphological diversity is observed across locations with divergent climates in a variety of species, such as extinct crocodylomorphs (relatives of extant Crocodylia)^18^. Inter-specific adaptation for smaller surface area to body size ratios in cooler climates (“Allen’s rule”), often achieved through reductions in limb length, is also widespread^16^,^19^. Intriguingly, intra-species variation in limb form in individuals from thermally divergent locations has also been observed in lizards but has yet to be explained^8^. Phenotypic differences seen in the North American lizard *Sceloporus occidentalis* include longer limbs relative to body size in individuals from warmer locations, and a reduction in relative limb length in cooler, more northern populations. Like many reptilian species, *Sceloporus* is not incubated by a parent and its incubation conditions are determined by the environment. Incubation temperature has previously been shown to influence embryonic motility and skeletal growth in chickens^20^. Changes in embryonic limb bone growth induced by pharmacologically stimulating embryo motility alone suggest that mechanical input, rather than temperature per se, regulates skeletal growth in embryonic limbs^21^,^22^,^23^,^24^,^25^.

Herein, we explore the hypothesis that developmental plasticity during prenatal stages of ontogeny allows for variation in limb proportions to emerge via environmentally-triggered alterations in movement. This hypothesis was tested in experiments using embryonic crocodiles (*Osteolaemus tetraspis* Cope 1861) and chickens (*Gallus gallus*). Previous studies in embryonic chickens indicate that altered incubation temperature modulates motility and limb growth^20^. This species uses contact incubation by a parent, however, and many birds alter their behaviours in different climates to maintain relatively constant incubation temperatures. Incubation at different temperatures within chickens is thus less likely to occur naturally^26^,^27^. Use of crocodiles, which are ectothermic, oviparous and not directly incubated by a parent, in contrast, provide an opportunity to explore whether motility and limb proportions are modified by incubation temperature in a model where such thermal variation could occur naturally. We specifically tested whether West African Dwarf crocodile embryos incubated at the extremes of the normal range for this species (32 °C and 28 °C) exhibited alterations in motility and limb proportions; we therefore made measurements of body size, limb length and stylopod, zeugopod and autopod element length. The relatively small number of eggs produced in a single *Osteolaemus* clutch limited our experiment to a single time-point, and did not allow for optimization of pharmacological manipulation of embryo movement in this species to separate the effects of temperature and embryo motility on limb growth.

Embryonic chickens, a commonly used model for mechanistic studies in limb development in which methods for pharmacological alteration of motility are well established and for which species-specific reagents are readily available^27^,^28^,^29^, were used to further explore our hypothesis. Specifically, we tested whether pharmacological immobilization alone alters limb proportions, when temperature is maintained constant to remove any influence it may have on embryo movement as a variable. Accordingly, limb element lengths were measured at selected time-points during development and their growth monitored in some experiments by repeat MRI imaging of individual control and immobilised chicken embryos to examine whether pharmacologically-induced alterations in movement alone produced modifications in limb proportions.

During early limb development, individual skeletal elements are laid down as cartilage condensations. Limb proportions emerge as a result of the size of the initial condensation^28^ and primarily due to later changes in their growth by endochondral ossification^5^, the process by which limb skeletal elements grow longitudinally^29^. Each element forms initially as a cartilage model that elongates via a controlled process of chondrocyte proliferation, maturation and hypertrophy before replacement by bone during ossification^30^,^31^,^32^. Muscle contraction has been implicated as a regulator of embryonic long bone growth by endochondral ossification^25^,^33^,^34^,^35^,^36^, but the molecular and cellular targets of such regulation have not been fully characterised. Use of the embryonic chick as a model allowed us to identify the mechanisms which underpin regulation of limb proportions by embryonic movement by examining the impact of altered embryo movement on both cellular behaviour and gene expression in individual growth plates. Measurements of growth plate zones widths, the expression of proliferative markers in the growth plate were made, and the size of hypertrophic chondrocytes from control and immobilised limbs was used to investigate the primary effect of embryo immobilisation on longitudinal growth. Unbiased array profiling was then used to evaluate gene expression in control and immobilised femur and tibiotarsus growth plates at selected time points during development. Using an inducible method for embryo immobilisation along with longitudinal tracking of limb growth has allowed us to investigate, and separate, changes in gene expression which are associated with determining the capacity of a growth plate cartilage to respond, sense and coordinate a subsequent growth response to mechanical cues. Our findings provide evidence that environmental factors, and not only genetic pre-specification may differentially influence limb element growth during prenatal development and thereby introduce phenotypic variation, which provides a novel insight into the potential evolutionary importance of developmental plasticity.

## Results

### Temperature influences embryonic crocodile limb proportions

**Figure 1:**
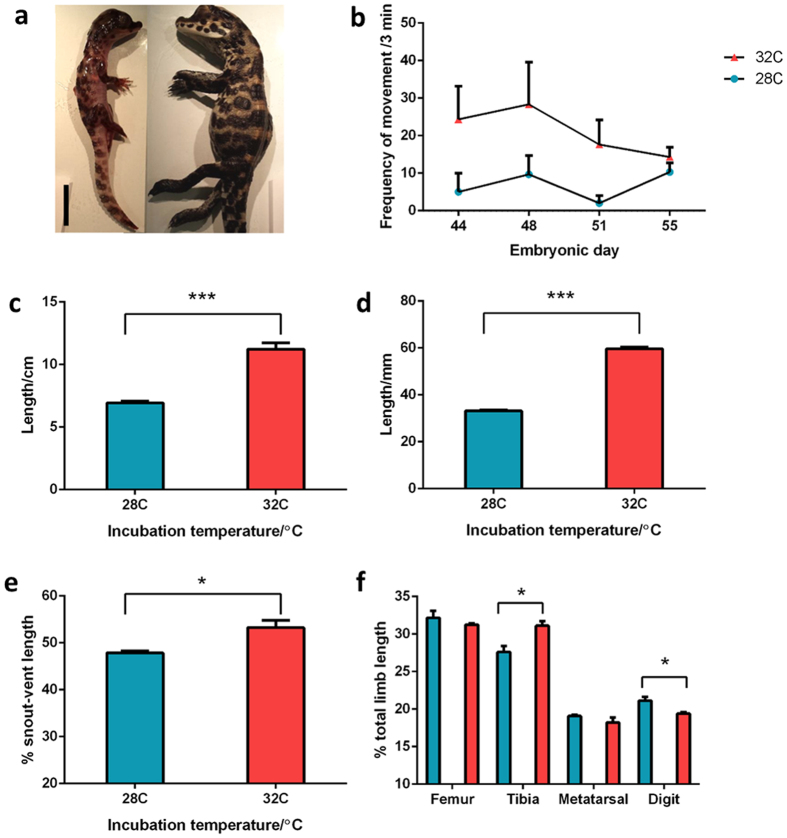
Motility and growth of West African Dwarf crocodile (*Osteolaemus tetraspis*) embryos incubated at 28 °C or 32 °C (n = 3 per group) and euthanised at E70, approximately hatching age. (**a**) Individuals from the 28 °C (left) and 32 °C (right) groups, demonstrating large differences in growth. Scale bar = 20 mm. (**b**) Number of embryo movements per 3 minute monitoring period during mid- to late incubation. (**c**) Body (snout to vent) length upon euthanasia. (**d**) Limb length. (**e**) Relative limb length (% snout-cloaca length). (**f**) Limb element lengths (% total limb length). Values are shown as mean ± SEM and were analysed using Welch’s t-test. *Indicates P < 0.05, ***indicates P < 0.001.

We investigated whether temperature alters motility and limb growth in the West African Dwarf crocodile. Embryos were incubated at 28 °C and 32 °C from embryonic day (E) 10–70. Embryo motility was monitored at E44, 48, 51 and 55. Snout-vent length, total limb length and total limb and limb element lengths corrected against body size were measured from embryos euthanised at E70. Embryos incubated at 28 °C were less motile when monitored at stage E51, close to mid-gestation (88% decrease in frequency of movement, *P* < 0.05, Fig. 1). The relatively heightened activity of embryos incubated at 32 °C persisted up until E55, when their large body size may have restricted *in ovo* movement.

Total body size at E70, around hatching age, was significantly smaller in embryos incubated at lower temperatures (38% smaller P < 0.05). Staging of embryos according to Ferguson’s^37^ staging series revealed that embryos incubated at 28 °C corresponded to stage 27 and E60–63, while those incubated at 32 °C corresponded to stage 28, which corresponds to E64–70 (with E70 considered hatching age). Staging at these late developmental stages is not based on morphological parameters but rather relative absorption of the yolk sac. A reduction in overall limb length was also observed (38% shorter snout-to-vent length and 44% reduction of limb length, *P* < 0.001 and 0.0001). Critically, the lower incubation temperature did not generate a uniform deficit in growth, where all elements might be expected to be affected equally, but rather disproportionately affected the length of specific skeletal elements over others, to change relative limb proportions. The hindlimbs in less motile embryos incubated at 28 °C were shorter when corrected for overall body size (10%, *P* < 0.05) and exhibited disproportionately shorter tibiae (11%, *P* < 0.05) but longer digits (9%, *P* < 0.05) compared to incubation at 32 °C. Examination at euthanasia revealed that all embryos were female, which rules out the possibility of sexual dimorphism in growth between temperature groups.

### Embryo movement is responsible for altered limb proportions

Our data from crocodile embryos indicate that some limb regions may be more severely affected than others, but did not establish whether this reflected temperature *per se* or altered movement. Chicken embryos were used for immobilisation studies, during which embryo movement was altered pharmacologically while constant 37 °C incubation temperature was maintained. Total limb and individual limb element lengths were measured in chicks immobilised between E10–14, 13–17 and 15–18 and compared to controls. Additionally, total limb and limb element lengths were measured in embryos immobilised from E10 onwards by daily MRI imaging between E14–18. Despite the onset of overt movement from E4.5 in the chick embryo, and “coordinated” movement from E7 onwards^38^, micro-CT-based measurements showed that total limb growth was unchanged by immobility imposed between E10–14.

**Figure 2:**
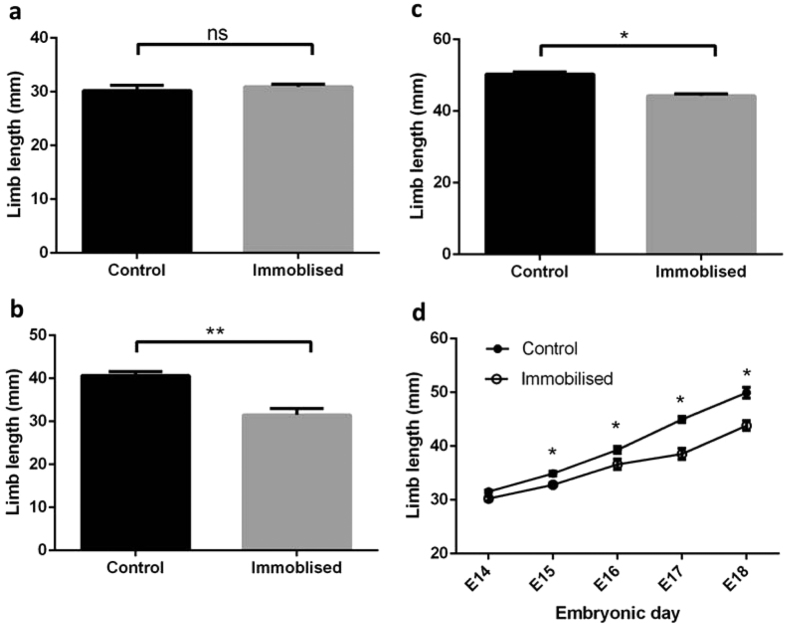
Total hindlimb length is reduced by removal of embryo movement at selected time-points during development. Mean ± SEM total limb length in control (n = 5) and immobilized (n = 4) chicken embryos is unchanged by (**a**) immobilization between E10–14. Total limb length is reduced by immobilization between (**b**) E13–16 and (**c**) E15–18 when compared by Mann-Whitney U-tests (*P* < 0.05 and 0.001 respectively). (**d)** MRI monitoring of mean ± SEM limb length daily in control (n = 6) and immobilized (n = 4) chicks. *Indicates P < 0.05.

**Figure 3:**
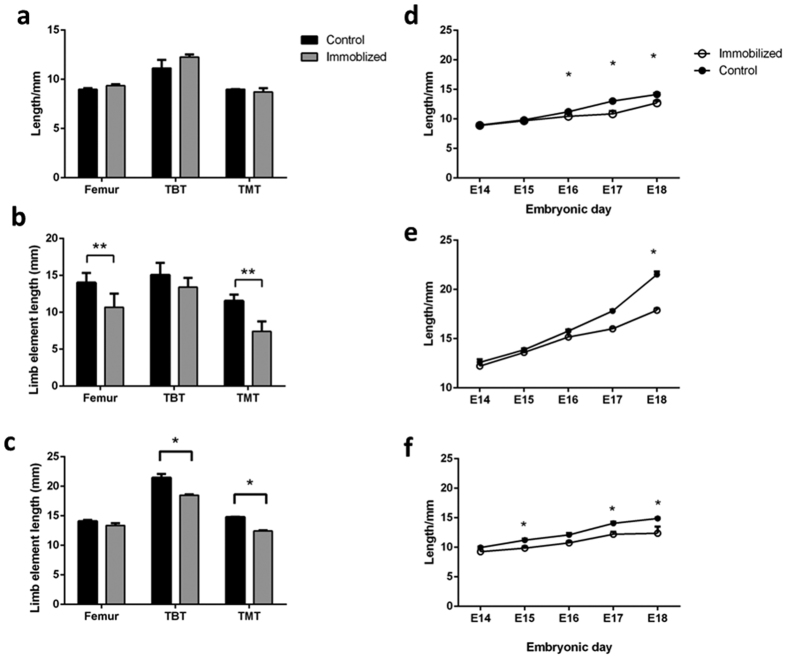
Limb proportions are altered by immobilization of embryonic chicks at selected time-points during development. Mean ± SEM lengths of femur, tibiotarsus (TBT) and tarsometatarsus (TMT) in control (n = 5) and immobilized (n = 4) chicken embryos is unchanged by (**a**) immobilization between E10–14. (**b**) Mean length of the femur and TMT but not TBT is reduced by immobilization between E13–16. (**c**) The length of all elements is reduced by immobilization between E15–18 when analysed by Mann Whitney U-test. MRI monitoring of mean ± SEM limb growth daily in control (n = 6) and immobilised (n = 4) chickens: (**d**) femur, (**e)** TBT and (**f)** TMT. *Indicates P < 0.5 and **indicates P < 0.01.

Pelvic limb length was reduced by the absence of movement only at later time points, between E13–16 and E15–18 (by 22% and 12% vs. vehicle-treated control, *P* < 0.001 and < 0.05 respectively; Fig. 2). Intriguingly, immobility between E13–17 significantly reduced both femur (24%, *P* < 0.001) and tarsometatarsus (TMT; 38%, *P* < 0.001) lengths while the tibiotarsus (TBT) was not significantly affected (Fig. 3). The TMT was most affected by immobilization between E15–18 (16% shorter compared to 12% in the TBT, *P* < 0.05), whereas in contrast the femur was not significantly reduced at this late time point.

MRI allows for non-invasive, longitudinal monitoring of chick development^39^,^40^. We used daily MRI scans to achieve repeat measures of limb growth between E14 to E18 in individual chicks immobilized from E10. These measures of limb and skeletal element length confirmed the existence of a period between E10–13 when lack of motility does not modify chick limb growth (see Fig. 2). Immobilisation between E15–18 achieved reductions in limb length without modification in eye diameter (a gross index of chick skull development^41^; note that HH-based staging after E13 relies on beak and 3^rd^ digit lengths which are both affected by immobilization. Such staging was thus not appropriate). We therefore staged only control chick embryos upon euthanasia after the final scan at E18 to assess whether cooling of eggs during MR imaging had an impact on development. Measurements of beak and 3^rd^ digit lengths from these chick embryos corresponded to those of E16 embryos^42^, indicating that these methods introduce a level of developmental delay. The altered limb proportions seen in MR-imaged embryos do however correspond closely with measurements made using microCT at E17; their altered limb proportions are consistent with selective modification of longitudinal growth by embryo movement.

Longitudinal MRI scans were used to explore the differing effects of chick immobilisation seen in individual limb elements at E13–17 and E15–18, and revealed that modifications in growth of each element in response to altered movement first emerge at different stages in each element. Longitudinal growth of the TBT showed significant reliance on movement only after E17, and the femur prior to E17, whereas TMT length was markedly modified by E15 (Fig. 3). Together these data indicate that different elements exhibit differential sensitivity to the removal of normal chick motility, which is seemingly unrelated to the proximo-distal sequence of limb element emergence.

### Growth plate dynamics are influenced by embryo movement

**Figure 4:**
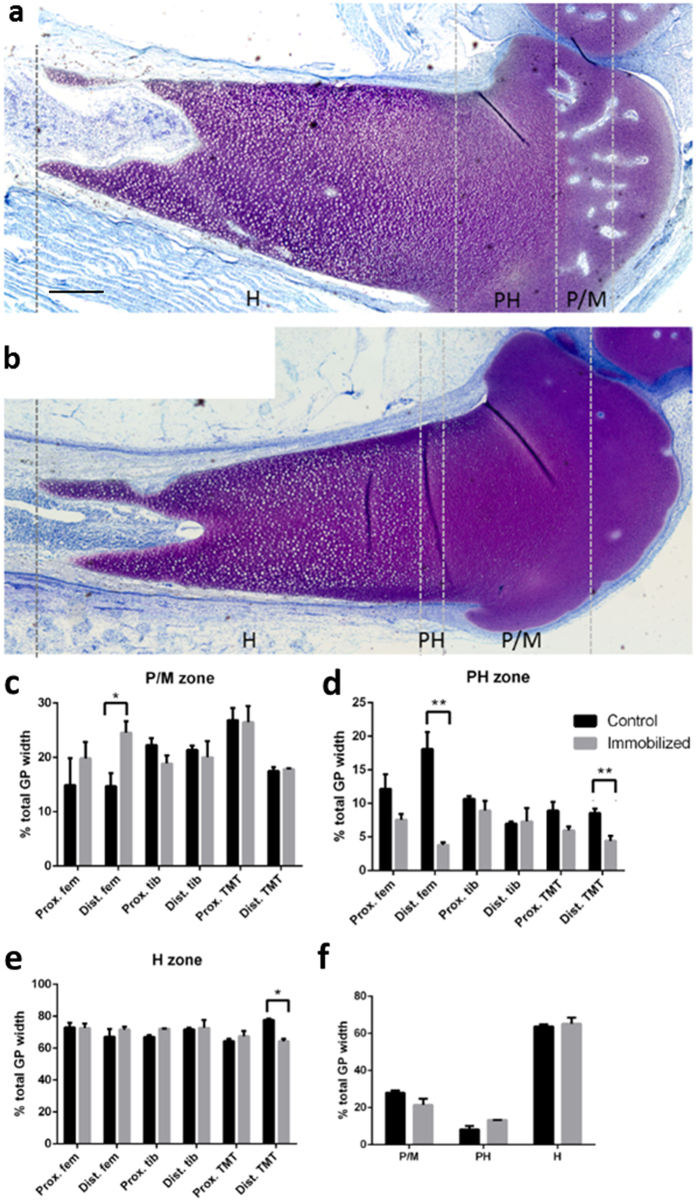
Altered embryo motility results in targeted changes in growth plate dynamics in the embryonic chick. (**a)** Growth plate of an embryonic day 18 (E18) control and (**b**) immobilized embryos (treated from E10 onwards) stained with toluidine blue. The proliferative/maturing zone (P/M), prehypertrophic (PH) and hypertrophic (H) zones are indicated by dotted lines. Quantification of average width ± SEM of (**c**) proliferative/maturing, (**d**) prehypertrophic and (**e**) hypertrophic zones as % total growth plate width in E18 control and immobilised limbs (n = 5 in each group). These were analysed by Mann-Whitney U-test. (**f**) Average width ± SEM of proliferative, prehypertrophic and hypertrophic zones in the distal femur of control & immobilised chick limbs (n = 4) at E14 shows a lack of modified growth plate dynamics at earlier stages. Values for control limbs are shown in black and immobilised in grey. Scale bar represents 200 μm. *Indicates P < 0.05, **indicates P < 0.01.

We next sought to relate the immobilisation-induced changes in limb proportions to modifications in the dynamics of specific growth plates. We quantified the widths of the distinct growth plate zones (proliferative/maturing, pre-hypertrophic and hypertrophic) in proximal and distal ends of the femur, TBT and TMT in sections from control and immobilized (from E10 onwards) chick pelvic limbs. Zone width was expressed as a percentage of the total growth plate width. Immobilization altered growth plate dynamics selectively in the distal region of both the femur and TMT, but not TBT, at E18 (Fig. 4). In contrast, the proximal regions of all elements were unaffected. Each growth plate in a given element does not contribute equally to longitudinal growth, and the changes we observed were targeted to the growth plate deemed responsible for >50% longitudinal growth^43^. No significant impact on either TBT growth plate was observed. Thus, differences in sensitivity to external input may be intrinsic to different limb regions.

**Figure 5:**
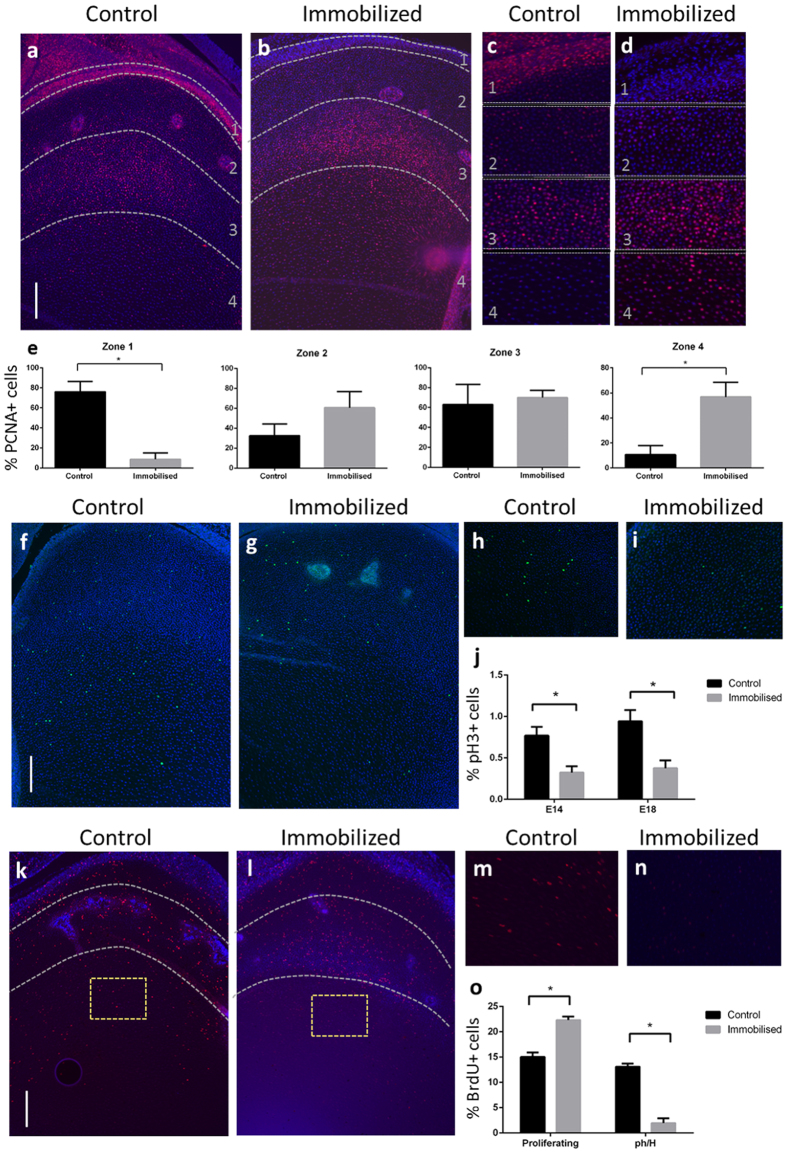
Proliferation in the embryonic chicken growth plate is disrupted by the removal of mechanical stimuli. PCNA expression in (**a**) control and (**b**) immobilized distal femur at E14. The mean ± SEM % total cells which express PCNA in the 1) articular cartilage, 2) “resting zone” cartilage, 3) proliferative zone and 4) prehypertrophic/hypertrophic zone was quantified in (**c**) control and (**d**) immobilised (n = 5 for each group) limbs at E14, after 4 days of treatment. (**e**) Mean ± SEM % total cells in each zone. Phosphohistone H3 expression in the (**f**) control and (**g**) immobilized distal femur at E14. The mean ± SEM % total cells that are phosphohistone H3 positive was quantified from a representative view of the epiphysis of (**h**) control and (**i**) immobilized limbs at E14, after 4 days of treatment, and E18, after 8 days of treatment (n = 5 in each group). (**j**) Mean ± SEM % total cells phosphohistone H3 positive. BrdU incorporation in (**k**) control and (**l**) immobilized E16 chick limbs sacrificed 4 hours after BrdU administration, after 6 days of treatment. The mean ± SEM % total cells which are BrdU positive in the proliferative zone (shown by dotted lines) or the prehypertrophic/hypertrophic zones of (**m**) control and (**n**) immobilised limbs (n = 5 in each group; indicated by yellow squares) 4 hours after BrdU administration was quantified. (**o**) Mean ± SEM % total cells phosphohistone positive in these regions. Mean values from the two treatment groups were analysed by Mann-Whitney U test. *Indicates P < 0.05. Scale bars represent 200 μm.

The impact of chick immobility in the affected growth plates at E18 was characterised by a relative expansion in the proliferative/maturing zone, with a corresponding narrowing of the pre-hypertrophic zone in femur and TMT (Fig. 5). We observed no impact of immobilization in any growth plate at E14 (Fig. 4f). These data suggest a correlation between modified growth plate dynamics and the changes in longitudinal growth of specific elements and limb proportions observed in immobilized chicks.

### Movement drives cellular progression in the growth plate

We tested the hypothesis that there is a correlation between chick embryo motility and changes in growth plate dynamics by examining cellular behaviour in the growth plate in response to immobilisation. We examined the regulation of chondrocyte proliferation, apoptosis and hypertrophy in the distal femoral growth plate of chicks immobilized from E10 onwards in comparison with control chick embryos. We compared the distribution of cells positive for proliferating cell nuclear antigen (PCNA, an S-phase marker) and phosphohistone H3 (a marker of mitosis), expressed as a percentage of the total number of cells (DAPI labelled).

Immobility from E10 produced a loss of PCNA labelling at the articular surface, little change in the proliferative zone and an increase in the number of PCNA positive cells in the prehypertrophic/hypertrophic zone, thus widening the region of PCNA labelling (normally restricted to the proliferative zone) by E14 (Fig. 5a–e). This was accompanied by a diminution in the number of phosphohistone H3-positive cells (Fig. 5f–j), indicating that the enlarged population of PCNA-positive cells do not complete the cell cycle and advance normally through the growth plate but rather accumulate. These changes were observed at E14, before any measurable impact of motility on femur length was evident, and were still apparent at E18 (see Supplementary Fig. S1).

Tracking of proliferating cells by BrdU incorporation in E16 chick limbs, 6 days after the onset of immobilization, confirmed that immobilisation slowed cellular progression through the growth plate; BrdU-positive cells in normal limbs had progressed to the prehypertrophic zone within 4 hours of BrdU administration, but were largely restricted to the proliferating zone in immobilised limbs (Fig. 5k–o). The rate of uptake of BrdU (cells per hour) was unchanged with immobilisation, suggesting that proliferative arrest in these cells occurs after completion of S-phase but prior to mitosis (Supplementary Fig. S2).

We found no alteration in number or distribution of TUNEL-positive cells in control and immobilized chick limbs at any time-point (Supplementary Fig. S3), indicating no impact on apoptosis. Measurement of chondrocyte cell area in regions of the hypertrophic zone adjacent to the chondro-osseous junction also showed no modification in hypertrophic expansion after 4 days of immobilization at E14. However, chondrocyte size was reduced by more prolonged lack of movement at E18 (21% reduction, *P* < 0.05, see Supplementary Fig. S4). These data indicate that movement initially targets specific growth plates by driving completion of the cell cycle within the proliferative zone.

### Molecular features of the growth response to mechanics

Our data suggest that longitudinal growth of embryonic limb elements is initially intrinsically regulated; “mechano-sensitive” limb growth is not always a feature of ontogeny but is acquired relatively late in development. Our data also suggest that, in the embryonic chicken, limb growth becomes responsive to altered embryo movement between E13–15 in the femur, but growth of the TBT is affected less severely by mechanics, and not until later time-points. This was exploited in an attempt to identify the molecular factors responsible for mechanical regulation of cartilage growth. Transcriptional profiling was performed to identify changes in coordinated gene expression associated with the switch from intrinsic to mechanical regulation of cartilage growth. Transcriptional profiles were compared in the distal femur and proximal TBT growth plates of control chicks at E12, 13 and 15 to explore whether changes in gene expression occur during the time period when femur growth becomes sensitive to movement but the growth of the TBT does not. Additionally, gene expression was compared in the distal femur and proximal TBT of chicks immobilised between E10–15 and compared to E15 controls, to explore whether any genes identified by the previous comparison were also regulated in expression by embryo movement.

**Figure 6:**
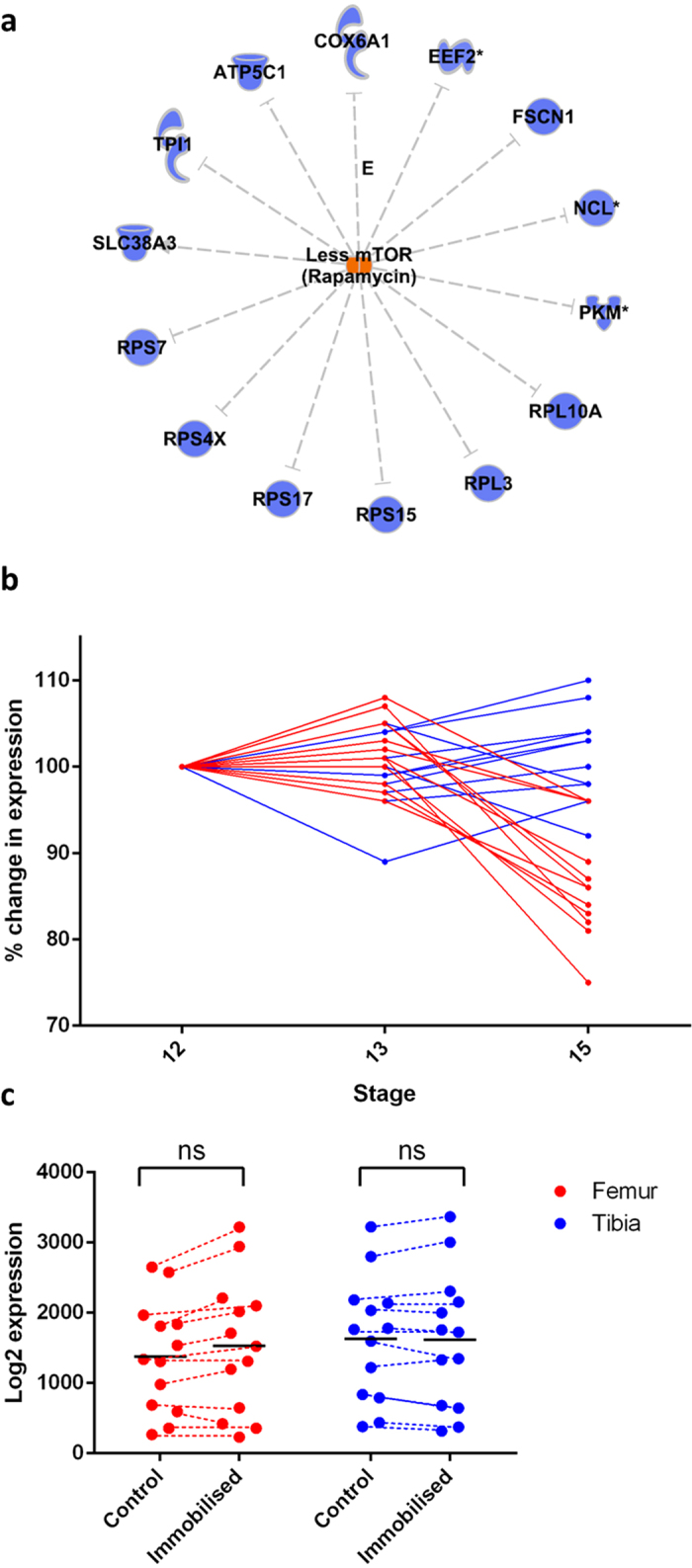
Differential expression of mTOR associated genes characterise femur and TBT elements at E15. (**a**) Upon analysis of E15 femur versus TBT (SAMR analysis), it was noted that 59 genes were differentially regulated between these limbs. These included ‘structural constituent of ribosome’ (p < 1E-08) and metabolic genes. Ingenuity up-stream analysis of this gene-list noted that less mTOR activity (p < 1E-09) was predicted in the femur, compared with the TBT (i.e. rapamycin being the inhibitor of mTOR) based on analysis of 54/59 genes in the database. mTOR regulated genes, shown in blue, are expressed at a lower level in E15 femur than E15 TBT. (**b**) The expression of these mTOR regulated genes was not significantly different between limbs at E12/13. Note that SLC38A3 expression is omitted from this graph due to its disproportionately higher expression level in the femur obscuring the other gene expression values. (**c**) Expression of the mTOR associated genes in the femur and TBT in growth cartilage in control and immobilised chicks. Expression patterns of these mTOR associated genes were unaltered by immobilisation. Femur data are in red and TBT in blue. Horizontal lines indicate mean expression values for all genes (n = 2–3 at each time point).

A comparison of control femur and TBT limb elements at E12 and E13 did not reveal any differentially expressed genes or pathways between these limb elements. Gene expression in pooled TBT and femur elements at E12 and E13 (to maximise statistical power) revealed a small number of modestly differentially expressed genes between the two time-points, 4 of which, namely Scube2, BTF3L4 and indirectly HMBG3 (via HMBG2) and IGF2BP3 (via links with IFG2), have known associations with endochondral ossification (Table 1). However, gene ontology (GO) analysis of these genes revealed enrichment of no distinct pathways or cellular components, suggesting this was a stochastic observation. Differences in expression specific to individual limb regions did, however, emerge by E15; 58 genes (4 upregulated and 54 downregulated) were found to differ in expression in the femur relative to the TBT at this time-point, and belong to specific biological pathways. GO analysis revealed enrichment of genes associated with Mechanistic Target of Rapamycin (mTOR) signalling among those downregulated specifically in the femur at E15 (Fig. 6a,b). The mTOR complex has diverse roles in the regulation of cell proliferation and growth and in our analysis appears to be the primary pathway altered between the femur and TBT elements at E15, suggesting it may regulate the emergence of limb proportions by specifically modulating the growth of those elements sensitive to movement.

**Figure 7:**
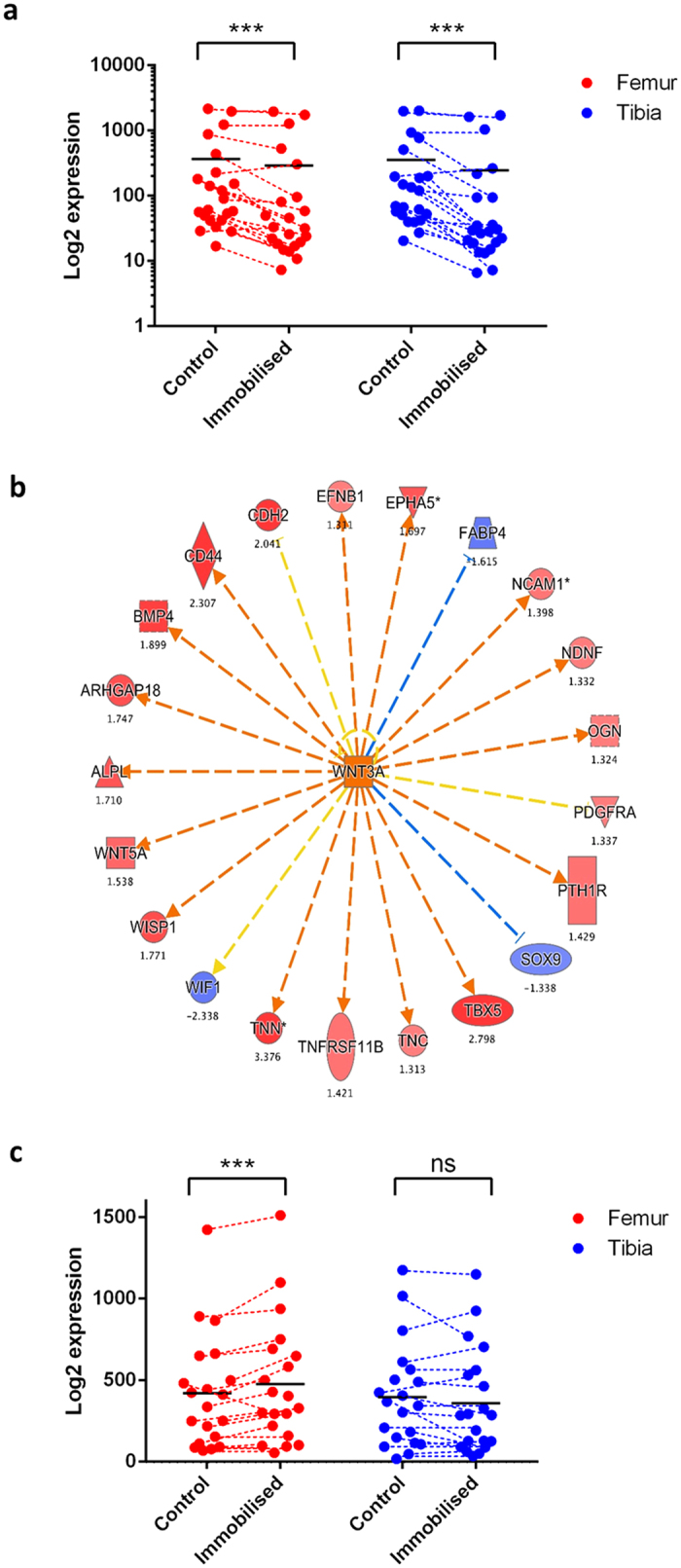
Gene expression in limb element growth cartilage in response to development and immobilisation. Analysis of changes in gene expression from E12/13 to E15 in both femur and TBT identified a robust expression of skeletal muscle genes at E12/13 followed by a strong down-regulation. Up-stream analysis of these genes identified that this included inhibition of MEF2c (p < 1E-10) (**a**) Plotting the 17 ‘myogenic’ genes directly associated with MEF2c finding, we noted these were further down-regulated during immobilisation in both the femur and TBT at E15 (plotting all muscle genes would have yielded a similar pattern). (**b**) Analysis of gene expression differences between DMB treated E15 femur and DMB treated E15 TBT (SAMR analysis) revealed that genes regulated by greater Wnt3a activity (Z = 2.1, p < 1E-10) and greater CTNNB1 (Z = 2.1, p < 1E-9) activity, were expressed at a greater level in the femur than TBT. Genes expressed at a higher level in DMB treated E15 femur are shown in red and lower-level are shown in blue. (**c**) The genes down-stream of Wnt3a are plotted using data from femur and TBT in growth cartilage from control and immobilised (DMB treated) chicks. Horizontal black lines indicate mean expression values for each gene.

We further sought to identify whether the genes which are associated with the acquisition of “mechano-responsive” cartilage growth are also involved in the mechano-sensory process, and in coordinating the growth response to mechanical stimuli. We compared gene expression in the femur and TBT elements in control and DMB-treated limbs at E15. The mTOR associated genes identified by the previous comparison were not significantly altered in their expression in either the femur or TBT in response to drug-induced immobilisation. In contrast, we found that a number of traditionally ‘myogenic’ genes associated with MEF2c were downregulated in both the femur and TBT in response to immobilisation (Fig. 7a). These genes are associated with actin-mediated cell contraction and have previously been implicated in the mechano-transduction process during embryonic limb growth^44^. However, although the skeletal muscle genes downregulated in response to immobilisation were very similar between limb elements, a number of genes associated with the regulation of chondrogenesis were altered in expression in the femur but not the TBT. Upstream analysis, which statistically links a combined pattern of up/down regulated set of RNAs with pre-existing signatures of drug or protein induced transcriptional responses^45^,^46^, identified that these changes are consistent with increased Wnt3a activity in the femur in response to immobilisation (Figs 5 and 7b,c). Our data suggest that genes associated with actin-mediated cell contraction are regulated in chondrocytes by embryo movement and may, as previously suggested^44^, contribute to sensing and transducing mechanical signals. Our data also suggest that Wnt3a is involved in coordinating chondrocyte responses to embryo movement in growth plate cartilage. However, such regulation of growth by movement only occurs in limb elements which have lower intrinsic mTOR pathway activity. Our analysis did not reveal which effectors of mTOR were responsible for the differences in intrinsic activity.

## Discussion

Our data provide evidence to support the hypothesis that the environment influences developmental pathways to produce phenotypic variation in limb form during embryonic development. Altered embryo behaviour and growth in response to incubation temperature in West African Dwarf crocodiles results in the emergence of hindlimb proportions that may, in turn, influence locomotor performance upon hatching, as well as survival in varying environments^9^,^12^,^47^. There are unfortunately no studies describing adaptive differences in extant crocodilian morphology to warmer or cooler environments. Nonetheless, similar correlation between limb length relative to body size and latitude (which is strongly linked to climate) has been observed in extant lizards^7^, suggesting that there is scope for such adaptive prenatal changes to occur in ectothermic, oviparous species. We observed that hind limbs in less motile embryos incubated at 28 °C were shorter when corrected for overall body size, with relatively shorter tibiae but longer digits compared to those incubated at 32 °C. Extant crocodilian embryos have been shown to scale limb proportions isometrically with body size after the very earliest stages of development, with negative allometry in the zeugopod (ulna) and positive allometry of the forelimb autopod providing the exceptions to this general isometric scaling^48^. A study which quantified the contribution of the femur, tibia and 3rd metatarsal to total limb length (digits were excluded) in *Alligator mississippiensis* from 8 days into incubation until hatching age (~70 days) showed that throughout prenatal ontogeny, the femur consistently contributes 43%, tibia 36% and 3rd metatarsal 21% to limb length, suggesting that their proportions do not to change^39^. Applying these methods of comparison to our data, we find that the values calculated from *Osteolaemus* embryos incubated at 32 °C are similar, with the femur contributing 40 ± 0.3%, tibia 39 ± 0.7% and metatarsal 21 ± 0.8% to total limb length. There are some differences in the values calculated from embryos incubated at 28 °C, in which the femur contributes 41 ± 1.1%, tibia 34 ± 0.9% and metatarsal 24 ± 0.9%. It remains to be established whether all autopod elements adhere to such isometric scaling during Osteolaemus ontogeny, but we speculate that it is unlikely that the differences observed in the relative length of the digits between embryos incubated at 28 °C and 32 °C can be explained solely on the basis that development is delayed.

The number of crocodile embryos available limited our study to a single time-point. To date, most studies into the embryonic development and limb scaling of crocodilians have focused on *Alligator mississippiensis*, likely due to their availability. Further studies to monitor limb growth throughout prenatal ontogeny in other species like *Osteolaemus* are required to fully understand how incubation temperature influences limb scaling in other crocodilians. However, the trend for isometric scaling of hindlimb proportions in *Alligator* suggests that the shorter tibiae and longer digits of *Osteolaemus* incubated at relatively cool temperatures may be due to differential regulation of limb growth at this temperature.

The observed reduction in hindlimb length relative to body size, and the proportional shortening of zeugopod and lengthening of autopod elements in crocodile embryos incubated at low temperatures, could be extrapolated to produce more “paddle-like” limbs in an extreme case. This has remarkable correspondence to limb adaptations to more aquatic environments observed in extinct crocodylomorphs (e.g. those adapted to more aquatic environments such as thalattosuchians show a trend for reduced zeugopod elements, including ulna, tibia and fibula^49^). The fossil record indicates that climate may have driven phenotypic diversity in crocodilian-line archosaurs, with less diverse, more terrestrial adaptations occurring at latitudes closer to the equator, while a greater range of diversity was associated with higher latitudes^18^. Correlation between morphology (i.e. limb length) and latitude/climate has also been reported in other reptiles and is reminiscent of Allen’s rule in mammals^7^,^8^,^50^. Direct regulation of cartilage growth by temperature has recently been proposed as a potentially comprehensive explanation for reduced extremity length and altered limb proportions in cooler climates^51^. However, direct temperature regulation of limb element growth does not fully explain the proportions we observe in the less motile crocodile embryos incubated at cooler temperatures. Although absolute growth of all elements was reduced at the lower 28 °C incubation temperature and a proportional reduction was seen in zeugopod length, the most peripheral autopod elements, which we would expect to be most severely ‘shortened’ by temperature regulation of cartilage growth, are instead proportionally elongated, rather than shortened. We suggest that mechanical regulation of embryonic bone growth also contributes to the variation in limb form seen in different climates by differentially regulating the growth of individual limb regions.

Pharmacological immobilization of embryonic chickens revealed that modulation in pelvic limb growth can be achieved by alteration of embryo motility alone, independent of incubation temperature. Our data suggest that this can be achieved via selective targeting of chondrocyte proliferation and progression in specific growth plates by embryo movement, to achieve changes in limb proportions. Our findings suggest that embryo motility acts to regulate limb proportions by exerting a differential impact on the growth of individual limb regions. This supports the findings of previous studies which showed that the impact of immobilisation on the growth of embryonic limb elements is complex, differs in individual limb regions and is partially dependent on the day at which immobilisation commences^23^,^24^,^35^,^36^,^52^. Previous studies by Lamb, *et al*.^36^ and Heywood, *et al*.^21^ which also reported an impact on limb length at E14 or later are consistent with this view. In contrast, Hosseini and Hogg^23^ reported an impact on femur and TBT/tibia length from E11 but did not observe any alteration in growth cartilage volume until 3 days later, at E14. Germiller and Goldstein^55^, on the other hand, showed an impact on element length and proliferation, without any changes in growth plate zone widths later than E11, from E13 onwards. Our use of 5μm resolution microCT to make measurements of element length found a significant impact of embryo movement only after E14. Despite examining limb growth in response to immobilisation across a very extensive period spanning E8–E20, these studies together appear to identify a comparatively short developmental period (potentially from E11 to E14) when sensitivity to movement commences. This variation may reflect inaccuracies introduced by the different methods used for measuring limb length, variation in the incubation temperature maintained between studies, the difficulty of accurately staging immobilised embryos (to allow for consistency) or even variation in growth rate between strains of domestic chicken used.

We observed that in limb elements which are not growing rapidly (e.g. the chicken femur between E15–18, Fig. 3), there is little scope to alter growth in response to immobilization. However, the differential impact of movement on each limb region cannot be fully explained by the region’s growth rate alone; e.g. the length of the TMT in chicks is severely affected by immobilization, even at developmental stages when other elements are growing more rapidly. Furthermore, our data suggest that “mechano-responsiveness” of longitudinal limb growth is not always a feature of ontogeny but is acquired during development. Our longitudinal data gathered from MRI analysis support the notion that different limb elements acquire mechanically-driven plasticity in growth at different stages. The developmental delay introduced by our imaging protocol means that we cannot ascertain exactly when mechano-sensitive growth is likely acquired in each limb element during normal chick development, but it certainly seems to differ within each of the individual stylopod, zeugopod and autopod limb regions. This developmental delay was ascertained by staging of control embryos after 4 days of MRI imaging, at E18, according to the Hamburger and Hamilton staging series^42^. Staging of DMB-treated embryos using this method was not possible as the growth of the traditional anatomical landmarks upon which the grading of late stages is based (beak and 3^rd^ digit length) is influenced by embryo immobilisation. However, we observed no difference in eye diameter (chosen as a gross index of skull development, as previous studies have identified that the growth of craniofacial bones is not influenced by embryo paralysis^54^) between control and immobilised embryos, which suggests that embryo paralysis does not cause an overall developmental delay but rather influences the growth of selected musculoskeletal elements. Investigating the impact of immobility on chick growth plate dynamics confirmed an impact of altered movement in the distal femur and TMT, but not in either TBT growth plate by E14, suggesting that differences in responsiveness to mechanical stimuli are instead intrinsic to different limb regions. The impact of altered motility on each individual element, however, differs between the chicken and crocodile species considered in this study.

Our analysis identified a population of cells in the proliferative zone of specific growth plates which progress slowly through the cell cycle in the absence of movement, and that cell cycle progression is accelerated to complete mitosis, and later differentiate, upon embryonic limb movement. A deficit in hypertrophic expansion, which appears to be secondary to the impact on proliferation, is also observed in the absence of normal mechanical input. The IGF-1-dependent phase of hypertrophy in particular is known to underpin the differential growth of limb elements in species which exhibit dramatic differences in limb proportions^56^; our data highlight that this may also be subject to regulation by movement depending on the intrinsic level of activation of canonical growth regulating pathways.

Transcriptional profiling by microarray analysis offers an explanation for why the growth of some elements is more severely affected by embryo immobilisation than others at the time-points chosen for this study. The inducible nature of pharmacological immobilisation of embryonic chicks, coupled with longitudinal monitoring of limb growth, allowed us to identify factors that determine whether a limb element is capable of responding to mechanics during prenatal growth. Previous embryo immobilisation studies have identified genes potentially involved in mechano-sensation and transduction and the response of embryonic limb growth to paralysis^44^. It was not possible in this study to separate the influence of spontaneous neuronal activity on gene expression from the consequent muscle activity that it stimulates. There are compounds which may be used to increase the frequency of embryonic movement, such as 4-aminopyridine, but these also provoke actions at the neuromuscular junction. However, the cytoskeleton-associated genes which we found to be altered in expression in immobilised limbs (Fig. 7a) have previously been implicated in chondrocyte mechano-transduction *in vitro*^57^,^58^, suggesting that this response at least is not dependent upon neuronal activity. Perhaps with the advent of newer methods for non-invasive imaging and monitoring of embryo movement *in ovo*^39^,^59^,^60^ it will be possible to better distinguish the contributions from muscle-induced mechanical stimuli from those induced by spontaneous neuronal activity.

Our analysis suggests that mechano-sensation and the growth response to mechanical stimuli can be de-coupled, and that additional factors exist which determine whether a growth response to mechanical stimuli will occur in individual limb regions. One such factor appears, on the basis of our transcriptional profiling of these elements, to be the intrinsic degree of mTOR pathway related activity.mTOR is a known regulator of the cell cycle and forms two multi-protein complexes known as mTORC1 and mTORC2, which perform different functions (Rokutanda, Fujita *et al*. 2009, Guan, Yang *et al*. 2014). mTORC1 is known to regulate protein synthesis in response to intrinsic factors such as nutrient and growth factor availability (Chen and Long 2014). The function of mTORC2 is less well characterised, but is also thought to regulate cellular metabolism through activation of Akt/PKB, and may play a role in cell adhesion or cytoskeletal organisation due to its stimulation of cytoskeletal-associated genes including paxillin and F-actin (Chen, Holguin *et al*. 2015). These complexes therefore function to integrate aspects of energy metabolism with the translation of mRNA during cell division. mTOR has previously been implicated as a regulator of chondrocyte proliferation and differentiation; the Akt-mTOR pathway positively regulates chondrocyte proliferation, maturation, matrix production and growth during skeletal development (Rokutanda, Fujita *et al*. 2009). There are, however, conflicting reports on the role of mTORC in the growth plate, with some studies reporting that inhibition of mTORC results in impaired chondrocyte proliferation, while others report only an impact on hypertrophy (Yang, Yang *et al*. 2013, Guan, Yang *et al*. 2014). These differences may reflect the relative impact on mTORC1 and mTORC2 signalling following mTOR inhibition. In our analysis, evidence for significantly lower mTOR pathway activity in the femur but not the TBT at E15 coincides with our findings showing onset of mechanosensitive growth in the femur only. These analyses also reveal a lack of any significant modification in transcriptional signature across E12 and E13 in the TBT and femur, suggesting that no marked change in their behaviour is likely to have occurred during this earlier timeframe. We propose that reduced mTOR-stimulated growth, likely to occur in response to other intrinsic factors such as nutrient availability, confers the ability for cells to be influenced by external, mechanical cues.

Activation of mTOR itself has also been shown by previous studies to be regulated by mechanical stimuli. Its activation is stimulated by cyclic loading in primary growth plate chondrocytes, and has been shown to be reduced in growth cartilage of pharmacologically immobilised chicks at E17 and in cases of postnatal limb immobilisation in humans^52^,^61^,^62^. However, while mTOR activation status appeared lower in the control femur relative to TBT at E15, the expression of genes associated with the mTOR pathway were not altered by immobilisation at E15 in our study; i.e. the difference between E15 femur and TBT was intrinsic to the femur and appears insensitive to gross changes in movement. This suggests that mTOR is likely not directly responsible for mechano-responsive growth. Rather, reduction in its activation in the femur may instead allow the influence of other factors, such as movement, to impact on growth. This is consistent with our observed changes in the expression of cytoskeletal genes associated with actin-mediated cell contraction with immobilisation in both TBT and femur, which previous studies have identified as a potential mediator in ‘*sensing*’ or transducing altered mechanical stimuli^44^. Our examination of changes in gene expression by immobilisation are also consistent with previous studies^44^ in that they suggest that the growth response to immobilisation is likely *coordinated* via Wnt3a activity. Indeed, these Wnt-related changes in expression occur in only the femur with immobilisation, consistent with Wnt’s proposed function in coordinating the growth response to embryo movement.

Thus, differential intrinsic mTOR activity in individual growth plates appears to help determine whether growth responds to external mechanical cues or is regulated only by intrinsic factors such as amino acid availability and growth factors. mTOR’s known regulation of protein synthesis during the cell cycle points to its potential to impact events in the growth plate proliferative zone. Our exploration of immobilisation-induced changes in the proliferative zone suggest that chondrocytes are arrested in the G2 phase of the cell cycle, which involves rapid protein synthesis in preparation for mitosis. Our observation that proliferative zone chondrocytes in the femur reach S phase at a normal rate (they incorporate BrdU during DNA replication) but fail to, or take longer to undergo mitosis strengthens this connection. We therefore propose that developmental downregulation of intrinsic mTOR activity results in reduced growth in the absence of embryo movement, and appears to allow the capacity for mechanical stimuli to instead drive cell cycle progression in the proliferative zone. Previous studies indicate that selection for intrinsic growth regulation can indeed occur at the expense of adaptability to mechanical loading^63^,^64^, although it is currently not clear how mTOR signalling would interact with Wnt3a mediation of the growth response (this need not be a direct interaction). Selection for different levels of intrinsic mTOR activity may offer an explanation for why different elements are more susceptible to growth regulation by mechanical stimuli in different species, such as the chicken and crocodile considered in this study.

In mammals, and to a lesser extent birds, the embryonic environment is controlled by a parent and, in the absence of pharmacological intervention or pathology, this appears to secure an optimal range of embryo motility to produce suitable limb forms, although at a major metabolic cost to the mother^65^. Many ectothermic oviparous species adopt a different reproductive strategy by producing larger numbers of eggs that are not incubated by a parent^66^. The incubation conditions of these eggs are dependent upon the environment and a single animal may produce multiple clutches of eggs in a season, and/or bury a single clutch of eggs in a nest containing a temperature gradient^67^, thereby increasing the chance that at least some embryos will experience ‘optimal’ incubation conditions. However, our data suggest that the reproductive strategy of these animals does not rely entirely on chance, but that developmental plasticity during the embryonic phase may also engender a robust capacity for the embryos of reptiles such as crocodiles (and perhaps other tetrapod species) to adapt their limb proportions in ways that may be beneficial in their post-hatching environment. Our findings also suggest that regulation of prenatal growth in response to embryo motility is achieved by the control of proliferation in limb growth plates which are specified by a mechanism involving intrinsic reduction in mTOR pathway activation.

## Materials and Methods

### Manipulation of embryo movement

All procedures complied with the Animals (Scientific Procedures) Act 1986 and were approved by the RVC animal welfare and ethical review body.

#### Crocodiles

Crocodile (*Osteolaemus tetraspis*) eggs from a single clutch were obtained from Crocodiles of the World conservation and education centre (Witney, UK) at E10. They were randomly assigned into groups and incubated at 28 °C or 32 °C (n = 3 per group) with 90% relative humidity. *Osteolaemus* are a threatened species not commonly bred in captivity, which limited the number of fertilised eggs available. Prior data indicate a small degree of variation in the limb element lengths of wild crocodilians sampled from the same geographical location^68^. Dupont and Plummer’s method for power and sample size calculations^69^ therefore indicate that the number of embryos required per group to detect a 10% change in limb element length with a power of 0.8 and type I error of 0.05 with varying temperature is ≥1. The chosen temperatures are within the ranges which produce viable, predominantly female hatchlings in captivity.

Embryo movement was monitored at intervals throughout incubation by candling the eggs and recording the number of visible movements during a 3 minute period^20^. Mean frequency of movement values were compared using Welch’s t-test. Embryos were euthanised at E70. Snout to cloaca length was measured with a tape measure and mean values compared by Welch’s t-test. The hindlimbs were removed and fixed in 4% paraformaldehyde.

#### Chickens

Fertilised eggs were incubated at 37 °C with 45% relative humidity. Eggs were ‘windowed’ on E4 using standard protocols^70^,^71^: briefly, a small section of shell and shell membrane were removed using sterile forceps and the shell was resealed with adhesive tape. Chicken embryos were immobilized with decamethonium bromide (DMB), which induces rigid paralysis^35^. Embryos were immobilized between E10–14, 10–18 13–16 and15–18. Sterile filtered 5 mg/ml DMB in 100 μl Tyrode’s solution was injected through the egg window onto the chorioallantoic membrane to induce immobilization. On each subsequent day, 100 μl of 1 mg/ml DMB in TS was administered to maintain paralysis. Embryos were monitored by viewing through the egg window on each day of treatment and any DMB-treated embryos which were not fully paralysed were excluded from the study. Control animals received equivalent volumes of TS. The embryos were euthanized on the final day of treatment, hindlimbs removed and fixed in 4% paraformaldehyde. The sex of chicken embryos used for this study was unknown, but previous studies indicate that sexual dimorphism in the growth of domestic chickens only occurs postnatally; body mass and growth hormone expression do not diverge between males and females until 2–4 weeks post hatching^72^,^73^.

### *Ex vivo* measurements of limb length from microCT scans

Limbs from crocodiles incubated at 28 °C or 32 °C (n = 3) and chickens treated with DMB or TS between E10–14, 13–16 and 15–18 (n = 5 per group) were stained with the contrast agent phosphotungstic acid (PTA) by immersing in a solution of 1% PTA 70% ethanol for 72 hours, to allow visualisation of cartilage by micro-computed tomography (microCT)^74^. Limbs were scanned with a SkyScan 1172 microCT system (Bruker, Belgium) with a resolution of 5 μm, voltage of 40 kv, current of 250 μA, exposure time of 1600 ms and a 0.5 mm aluminium filter. Images were reconstructed using NRecon and whole limb lengths and length of the femur, TBT and TMT elements measured using DataViewer software (Bruker, Belgium). Limb measurements from the different chicken and crocodile groups were compared using Mann-Whitney U-tests and Welch’s t-tests, respectively.

### Noninvasive monitoring of embryonic limb growth *in ovo*

Chicken embryos treated with TS (n = 6) or DMB (n = 4) between E10–18 were imaged daily between E14–18 with a Philips Intera 1.5 T MR system with a SENSE flex spinal coil (Philips Medical Systems). The eggs were cooled for 10 minutes at 4 °C prior to imaging to temporarily reduce embryo movement in TS treated chicks. The eggs were imaged in the dorsal plane using a fast field echo sequence with a flip angle of 60 °C, echo time of 3.41 ms and repetition time of 7.62 ms. Time out of the incubator for imaging did not vary between TS and DMB treated groups. The embryos were euthanized after the final scan on E18 and staged according to the Hamburger and Hamilton staging series^44^ to assess the impact that removing the eggs from the incubator daily and cooling had on embryonic development; staging was based on third digit and beak length, which were measured with digital callipers. The scan images were imported into ImageJ^75^ as image stacks and total limb length and element length were calculated by identifying the most proximal and distal bone points in the image stack^76^. Mann-Whitney U-tests were used to compare the DMB-treated embryo values to controls at each timepoint.

### Sectioning and growth plate histology

Fixed, dissected limbs from embryonic chicks treated with TS/DMB between E10–14 and E10–18 were processed to paraffin for embedding and sectioning. 6 μm sections were stained with Toluidine blue. Sections were imaged using a DM4000B upright microscope and DC500 colour camera, both controlled through Leica Application Suite software version 2.8.1 (all from Leica Microsystems, Milton Keynes, UK). The objective used was a 10x HC PL FLUOTAR PH1 (NA = 0.3). Growth plate zones (proliferating/maturing, prehypertrophic and hypertrophic) were identified based on cell morphology and organisation, measured and expressed as a proportion of the total growth plate width^77^ using ImageJ. All growth plate zones were identified and measured by the same observer, and the growth plate images were temporarily assigned a random ID number unrelated to treatment group during analysis to minimise bias. Mean growth plate widths were compared using Welch’s t-test.

### Assessment of cellular kinetics in the growth plate

We performed immunohistochemistry to detect proliferating cell nuclear antigen (PCNA) and phosphohistone H3 using sections from the distal femoral growth plate of embryonic chicks treated with TS/DMB between E10–14 and −18 (n = 5 per group, full method including antibody dilutions in Supplementary Methods). The epiphysis was divided into 4 zones to assess distribution of PCNA-labelled cells: the articular cartilage, “resting” zone, proliferating zone and prehyperophic/hypertrophic zone. The proportion of PCNA labelled cells to total DAPI labelled cells was calculated in each zone for PCNA, and in a representative view of the growth plate for phosphohistone H3. BrdU incorporation by proliferating cells was assessed in TS/DMB treated embryos at E16, in embryos euthanised 4 hours after BrdU administration (see Supplementary methods). We quantified the number of BrdU-positive cells, expressed as a proportion of total DAPI-labelled cells, in the proliferative zone and prehypertrophic/hypertrophic zones using Image J. Sections were imaged using a DM4000B upright microscope with samples illuminated using an EBQ100 light source and filter cubes A4, L5, and TX2 (all from Leica Microsystems, Milton Keynes, UK) and an AxioCam MRm monochrome camera controlled through Axiovision software version 4.8.2 (Carl Zeiss Ltd, Cambridge, UK). The objectives used were: 5x HC PL FLUOTAR (NA = 0.15); 10x HC PL FLUOTAR PH1 (NA = 0.3). Mean values from control and immobilized limbs were compared using Mann-Whitney U-test.

The size of chondrocytes from the distal femoral hypertrophic zone adjacent to the chondro-osseous junction was assessed in freshly killed chicken embryos treated with TS or DMB between E10–14 and E10–18 (n = 6 in each group). One femur from each chick was removed and bisected sagitally^78^. The tissue was maintained at 37 °C in standard culture media until required, then incubated with 5 μM calcein AM for 15 minutes at 37 °C to fluorescently label viable cells. A representative area from the base of the hypertrophic zone was then collected as an image stack in 3D using an SP5 confocal microscope using Leica Application Suite advanced fluorescence software version 2.6 (Leica Microsystems, Milton Keynes, UK). The objective used was a 40x HCX PL FLUOTAR PH2 (NA = 0.75). The average maximum cell area of calcein AM labelled hypertrophic chondrocytes was quantified from image stacks from representative regions from the hypertrophic zone in control and immobilized chicks ( > 20 cells per growth plate) using ImageJ and compared using Mann-Whitney U-test. Apoptosis was evaluated using TUNEL assay (see Supplementary Methods).

### RNA extraction and Microarray analysis

The hindlimbs were dissected from freshly killed embryos treated with either TS or DMB from E10 onwards. The growth cartilage from the distal femur and proximal TBT was dissected. Any fully ossified bone, bone marrow and red blood cells were excluded. The tissue was snap-frozen by immersing in liquid nitrogen and stored at −80 °C until required. The tissue was not allowed to thaw prior to RNA isolation. The following samples were collected: control femur and TBT RNA from E12, control femur and TBT RNA from E13, control femur and TBT RNA from E15, immobilised femur and TBT RNA from E15 (n = 3 biological replicates in each group. Each biological replicate contained the pooled growth cartilage from 4 individual chicken embryos.

Total RNA was isolated using TRizol^®^ (Life Technologies). RNA was dissolved in 20 μl RNAse-free water. Affymetrix DNA microarrays (Chicken) were processed according to the manufacturer’s protocol (BEA Core facility, Huddinge Hospital, Sweden). From the twenty four samples processed, 1 sample was excluded as an outlier by visually evaluating principal component analysis prior to down-stream analysis. The raw data has been deposited at the Gene Expression Omnibus (accession no.: GSE77412). Aroma.affymetrix^79^ and Bioconductor^80^ packages in R were used to preprocess the CEL files and generate summarized expression matrices. A standard deviation filter (SD = 10) was applied to remove low expressed invariant probe-sets (which reflect background signal or poorly functioning probesets) and this value is array type specific (obtained by first plotting the distribution of SD values). For annotation, the Chicken_Gg_ENST, binary.cdf was used. For comparing various groups an unpaired SAMR (significance analysis of microarrays) analysis was used to estimate the false discovery rate (FDR) using 5,000 permutations. The nearest FDR value to 5% was utilized in the pathway or ontology analysis. There are limitations to the usefulness of pathway or gene ontology (GO) analysis for identifying enrichments of biological functions among genes in a list of regulated genes^81^. Thus, to interpret a significant p-value for GO analysis as being evidence of specific enrichment, the ‘regulated gene list’ (in this case genes with AEU events) must be contrasted with a list of the *detectable* genes in the experiment (and not all the genes in the genome or on the gene-chip). Only when the experimentally regulated list contains enriched p-values well above those observed due to bias did we consider the observation reliable. As well as Ingenuity IPA analysis we utilized GOstats package in R with current GO categories (GO.db) to confirm the IPA analysis. Input gene lists, and related background expression gene lists were used in IPA and GO with an ~5% FDR and no-fold change filter. The latter is used because it is well established that a large number of coordinated but modest gene expression changes can meaningfully underlie shifts in pathway regulation.

## Additional Information

**Accession codes:** Microarray data has been deposited at the Gene Expression Omnibus (accession no.: GSE77412).

**How to cite this article:** Pollard, A. S. *et al*. Limb proportions show developmental plasticity in response to embryo movement. *Sci. Rep.* **7**, 41926; doi: 10.1038/srep41926 (2017).

**Publisher's note:** Springer Nature remains neutral with regard to jurisdictional claims in published maps and institutional affiliations.

## Supplementary Material

Supplementary Information

## Figures and Tables

**Table 1:** Genes differentially expressed between control femur and control TBT at E12/13, resulting from unpaired significance analysis of microarrays analysis (SAMR), with ~5% false discovery rate (FDR).

Gene Symbol	Gene Name	Fold Change	Known association with endochondral ossification
RAB10	RAB10, member RAS oncogene family	1.179574992	
NRIP3	nuclear receptor interacting protein 3	1.242047415	
SCUBE2	signal peptide, CUB domain, EGF-like 2	1.14502171	Regulates endochondral ossification, likely via IHH^82^
ACTC1	actin, alpha, cardiac muscle 1	1.502716256	
HMGB3	high mobility group box 3	1.102570551	Closely related HMBG2 is expressed by relatively undifferentiated chondrocytes with roles in chondrocyte differentiation and articular cartilage via beta-catenin pathway^85^
OCM2	oncomodulin 2	1.728998125	
BLMH	bleomycin hydrolase	1.139048505	
BZW2	basic leucine zipper and W2 domains 2	1.085036637	
SRL	sarcalumenin	1.316752616	
SRP68	signal recognition particle 68 kDa	1.099992098	
BTF3L4	basic transcription factor 3-like 4	1.148606842	Associated with chondrocyte differentiation^88^
IGF2BP3	insulin-like growth factor 2 mRNA binding protein 3	1.080702446	Chondrocyte differentiation^89^,^90^
Unknown	Hypothetical protein	1.368471921	
MAP1B	microtubule-associated protein 1B	1.301912506	
Unknown	Hypothetical protein	1.390719923	

These 17 genes were differentially expressed between control femur and control TBT at E12/13but failed to show enrichment in any specific pathway or biological function using Ingenuity IPA analysis and GOstats package in R with current GO categories (GO.db) for confirmation. Input gene lists, and related background expression gene lists were used in IPA and GO with an ~5% FDR and no-fold change filter.
