# Supplementary material for: Limb proportions show developmental plasticity in response to embryo movement
Source: Sci Rep. 2017 Feb 6;7:41926. doi: 10.1038/srep41926 (PMC5292730; doi:10.1038/srep41926)
Supplement: Supplementary Information [file srep41926-s1.pdf]

Limb proportions show developmental plasticity in response to embryo movement

A.S. Pollard[1*], B.G. Charlton[1], J.R. Hutchinson[1], T. Gustafsson[2], I.M. McGonnell[1], J.A. Timmons[3] and A.A. Pitsillides[1]

SUPPORTING INFORMATION

Supplementary Methods

Assessment of proliferation

We performed immunohistochemistry to detect proliferating cell nuclear antigen (PCNA), a marker of S phase, and phosphohistone H3, a marker of mitosis, using 6µm paraffin sections from the distal femoral growth plate of embryonic chicks treated with TS/DMB between E10-14 and -18 (n=5 in each group). PCNA was detected using mouse anti-PCNA antibody (Abcam) at a 1:200 dilution in PBS followed by AlexaFluor 594 goat anti-mouse secondary antibody at a 1:500 dilution in PBS (Life Technologies) and phosphohistone H3 was detected using rabbit anti-phosphohistone H3 (Cell Signalling Technology) antibody at a 1:500 dilution followed by AlexaFluor 488 goat anti-rabbit secondary antibody (Life Technologies) at 1:500 dilution. No antigen retrieval was necessary for PCNA labelling, but retrieval by boiling sections in Tris-EDTA buffer (10mM Tris Base, 1mM EDTA, 0.05% Tween 20, pH 9) for 10 minutes was necessary for phosphohistone H3 labelling. Sections were mounted using ProLong Gold Antifade mounting agent containing DAPI (Life Technologies). The epiphysis was divided into 4 zones to assess the labelling frequency and distribution of PCNA-labelled, relative to DAPI-labelled, cells: the articular cartilage at the joint surface, “resting” zone chondrocytes, the proliferating zone and the prehyperophic/hypertrophic zone. The proportion of positively labelled cells to total cells labelled with DAPI was calculated in each of the above zones for PCNA, and in a representative view of the whole epiphysis for phosphohistone H3.

BrdU incorporation by proliferating cells was assessed by treating embryos *in ovo* (n=5) at E16 with 50µl 5mg/ml BrdU by injection onto the chorioallantoic membrane. Embryos were sacrificed 4 hours after BrdU administration and the distribution of BrdU positive cells was assessed in paraffin sections from the distal femoral growth plate. This required denaturation of DNA by incubating sections in 2N HCl at 37°C for 30 minutes followed by immersion in borate buffer for 10 mins at RT. BrdU was detected using biotinylated mouse anti-BrdU antibody (Pierce Antibodies) at 1:500 dilution followed

by Alexa Fluor 594-conjugated streptavidin (Life Technologies) at 1:500 dilution. Sections were mounted using ProLong Gold Antifade mounting agent containing DAPI (Life Technologies). To assess how immobilization influences progression from the proliferative zone, we quantified the number of BrdU-positive cells, expressed as a proportion of total DAPI-labelled cells, in the proliferative zone and prehypertrophic/hypertrophic zones using Image J. Mean values from control and immobilized limbs were compared using Mann-Whitney U-test.

Assessment of apoptosis

Apoptosis in the distal femoral growth plate was assessed in paraffin sections from chicken embryos treated with TS or DMB between E10-14 and E10-18 (n=5 in each group) using TUNEL assay to detect fragmented DNA. A DeadEnd Colorimetric TUNEL assay kit (Promega, Southampton, UK) was used according to the manufacturer's instructions. Positive controls were included by treated sections with DNAse I in 50mM Tris-EDTA pH 7.5 with 1mg/ml bovine serum albumin in PBS for 10 minutes at RT prior to the labelling procedure. Negative controls were included by incubating sections with biotinylated nucleotides only, omitting the terminal transferase enzyme.

Supplementary Figures

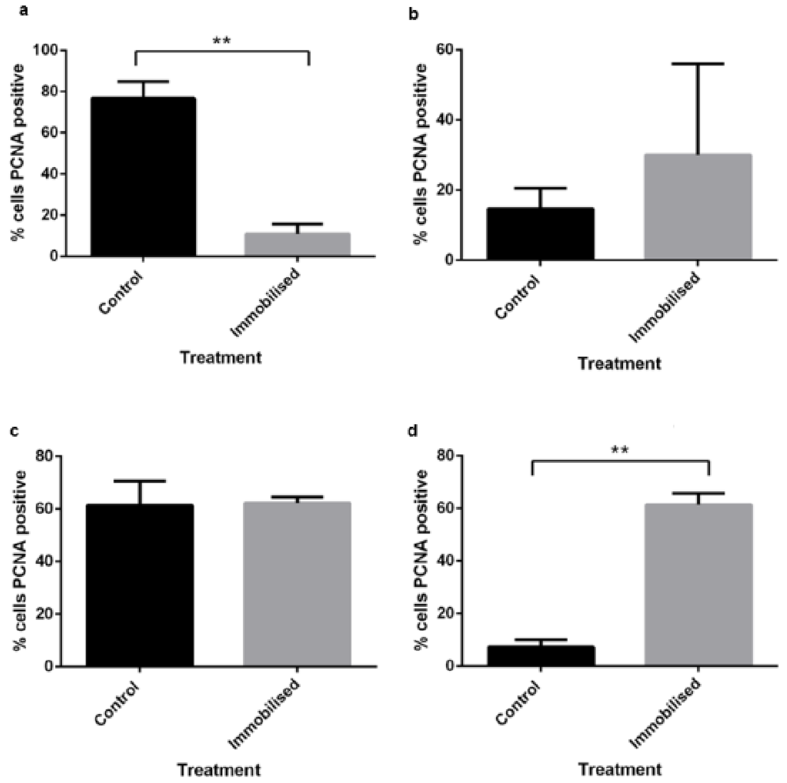

Fig. S1. Distribution of cells expressing PCNA in the distal femoral growth plate at E18, 8 days after onset of immobilisation. The distribution of PCNA-positive cells is unchanged from E14, 4 days after the onset of immobilisation. The proportion of PCNA-positive cells (mean ±SEM) is expressed as a percentage of total in A) the articular cartilage, B) resting zone, C) proliferative zone and D) prehypertrophic/hypertrophic zone in control and immobilised limbs (n=4 for each group). A similar distribution of PCNA-positive cells in the growth plate is observed at E14, after only 4 days of immobilisation.

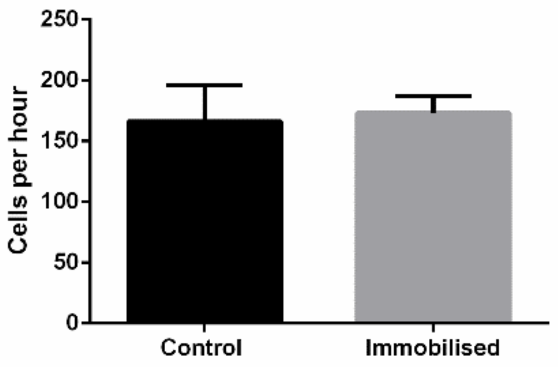

Fig. S2. Rate of incorporation of BrdU (expressed as mean ±SEM no. cells per hour) by proliferating cells in the distal femoral growth plate at E16. The rate at which cells reach S phase and incorporate BrdU during DNA replication is unchanged between control and immobilised embryos (n=5 in each group).

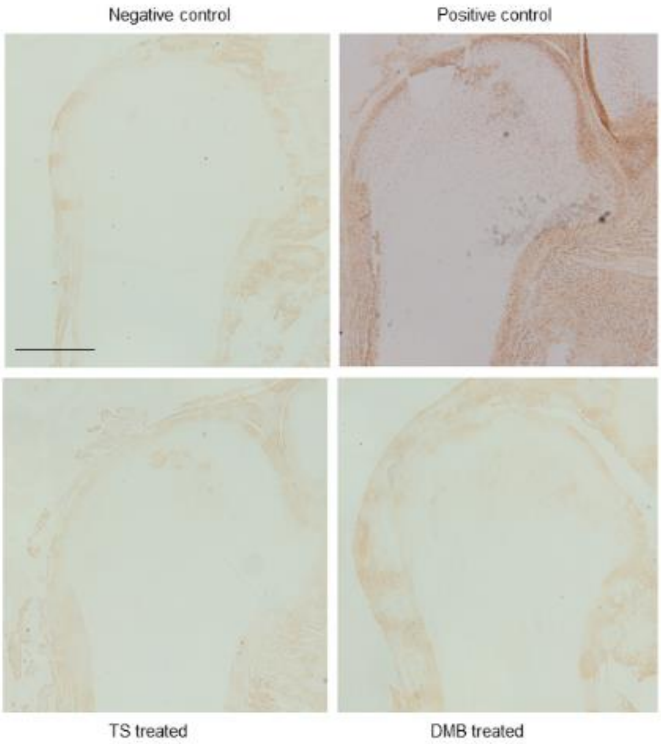

Fig. S3. Identification of apoptotic cells by TUNEL assay in the distal femoral growth plate of control and immobilised E14 chick limbs. Minimal apoptosis was detected in either control (n=5) or immobilised (n=5) embryonic growth plates, indicating that apoptosis is unlikely to be regulated by embryo movement. Positive (DNAse I treated) and negative (terminal deoxynucleotidyl transferase enzyme omitted) controls were included to validate the method. Scale bar represents 500µm.

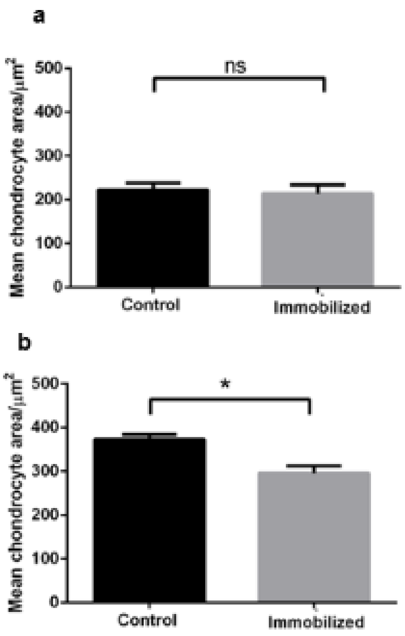

Fig. S4. Immobilisation of embryonic chickens results in a deficit in hypertrophic expansion. Average maximum cell area ±SEM was quantified in representative regions from the distal femoral growth plate hypertrophic zone in control and immobilized chicks (n=5/6 in each group, >20 cells per growth plate). A significant reduction in cell area was seen 8 days after onset of immobilization, at **B**) E18 (P<0.05) but not at **A**) E14 (P=0.75).

Gene Symbol	Gene Name	Fold Change	Known role in endochondral ossification/chondrogenesis
DPT	Dermatopontin	1.8	Cartilage extracellular matrix component [1]
RECK	Reversion inducing cysteine rich protein with kazal motifs	1.3	Limb patterning and chondrocyte differentiation [2,3]
WEE1	Wee1-like protein kinase	1.3	Cell cycle regulation[4]
PDGFRL	Platelet derived growth factor receptor like	1.2	PDGF involved in bone growth during development[5]
RPS7	RPS7 ribosomal protein S7	-1.1	Protein synthesis role. Highly expressed in articular cartilage[6]
CKAP4	Cytoskeleton-Associated Protein 4	-1.1	
EEF2	Eukaryotic Translation Elongation Factor 2	-1.1	Protein translation role. Synthesis is influenced by mechanical load in chondrocytes [7,8]
Cu		-1.1	
RPL4	Rpl4 ribosomal protein L4	-1.1	
NCL	Nucleolin	-1.1	
PLOD2	procollagen-lysine,2-oxoglutarate 5-dioxygenase 2	-1.1	Expressed during chondrocyte differentiation, involved in collagen type I and II synthesis[9]
HNRNPA3	Heterogeneous Nuclear Ribonucleoprotein A3	-1.1	
AKIRIN2	Akirin-2	-1.1	
RPL3	Ribosomal Protein L3	-1.1	

ATP6	ATP synthase subunit 6	-1.1	
RPS11	Ribosomal Protein S11	-1.2	
ENSGALG00000000489		-1.2	
ENSGALG00000026335		-1.2	
RPL10A	Ribosomal Protein L10a	-1.2	
PRRC2C	Proline Rich Coiled-Coil 2C	-1.2	
DDOST	Dolichyl-Diphosphooligosaccharide--Protein Glycosyltransferase Non-Catalytic Subunit	-1.2	Expressed in embryonic articular cartilage[10]
DKC1	Dyskerin	-1.2	Expressed in growth plate proliferative zone[11]
ATP5H	ATP Synthase, H+ Transporting	-1.2	
RPS4X	Ribosomal Protein S4, X-Linked	-1.2	Highly expressed in human fetal cartilage[12]
TMEM101	Transmembrane Protein 101	-1.2	
ATP5C1	ATP Synthase, H+ Transporting, Mitochondrial F1 Complex, Gamma Polypeptide 1	-1.2	
ASB9	Ankyrin Repeat And SOCS Box Containing 9	-1.2	
TPI1	Triosephosphate Isomerase 1	-1.2	
FSCN1	Fascin Homolog 1, Actin-Bundling Protein	-1.2	
XYLT1	Xylosyltransferase 1	-1.2	Proteoglycan synthesis during cartilage matrix production[13]
GAPDH	Glyceraldehyde-3-Phosphate Dehydrogenase	-1.2	

Enpp2	Ectonucleotide pyrophosphatase/phosphodiesterase 2	-1.2	
RPLP1	Ribosomal Protein Lateral Stalk Subunit P1	-1.2	
RPS15	Ribosomal Protein S15	-1.2	
ENSGALG00000009839		-1.2	
NDUFB9	NADH:Ubiquinone Oxidoreductase Subunit B9	-1.2	
ATP6V0C	ATPase H+ Transporting V0 Subunit C	-1.3	
TCEB3	Transcription Elongation Factor B Subunit 3	-1.3	
TMF1	TATA Element Modulatory Factor 1	-1.3	
Mtch2	Mitochondrial Carrier 2	-1.3	
CIB1	Calcium And Integrin Binding 1	-1.3	
SLC16A1	Solute Carrier Family 16 Member 1	-1.3	
COX6A1	Cytochrome c oxidase subunit 6A1	-1.3	
SLC16A3	Solute Carrier Family 16 Member 3	-1.3	
VCP	Valosin Containing Protein	-1.3	
UQCR10	Ubiquinol-Cytochrome C Reductase, Complex III Subunit X	-1.4	
LMF2	Lipase Maturation Factor 2	-1.4	
PAPSS2	3'-Phosphoadenosine 5'-Phosphosulfate Synthase 2	-1.4	Sulphation of proteoglycans in growth plate cartilage, expressed in proliferative zone[14]

PKM	Pyruvate Kinase, Muscle	-1.4	
RPS17L	Ribosomal Protein S17	-1.4	
COX5A	Cytochrome C Oxidase Subunit 5A	-1.4	
PPIB	Peptidylprolyl Isomerase B	-1.4	Mutations result in osteogenesis imperfecta, interacts with procollagen and likely influences collagen I structure
SLC38A3	Solute Carrier Family 38 Member 3	-1.4	
SFRP1	Secreted Frizzled-Related Protein 1	-1.5	Regulator of Wnt signalling in chondrocyte differentiation[15]
RSPO3	R-spondin 3	-1.5	Activator of Wnt/beta-catenin signalling with potential role in chondrocyte intercalation[16]
PTTG1IP	Pituitary Tumor-Transforming 1 Interacting Protein	-1.5	Target of Runx2, which is a regulator of osteoblast development and chondrocyte maturation[17]
PTCH2	Patched 2	-1.8	Involved in hedgehog signalling during embryogenesis, not specifically chondrocytes [18]

Supplementary Table S1. Genes differentially expressed between control femur and control TBT at E15, resulting from significance analysis of microarrays analysis, with ~5% false discovery rate (FDR). Input gene lists, and related background expression gene lists were used in IPA and GO with an ~5% FDR and no-fold change filter

Supplementary References

1 Derfoul, A., Perkins, G. L., Hall, D. J. & Tuan, R. S. Glucocorticoids Promote Chondrogenic Differentiation of Adult Human Mesenchymal Stem Cells by Enhancing Expression of Cartilage Extracellular Matrix Genes. *STEM CELLS* **24**, 1487-1495, doi:10.1634/stemcells.2005-0415 (2006).

2 Yamamoto, M. *et al.* The transformation suppressor gene Reck is required for postaxial patterning in mouse forelimbs. *Biology open* **1**, 458-466 (2012).

3 Kondo, S. *et al.* Dual effects of the membrane-anchored MMP regulator RECK on chondrogenic differentiation of ATDC5 cells. *Journal of cell science* **120**, 849-857 (2007).

4 Tran, T., Kolupaeva, V. & Basilico, C. FGF inhibits the activity of the cyclin B1/CDK1 kinase to induce a transient G2 arrest in RCS chondrocytes. *Cell Cycle* **9**, 4379-4386, doi:10.4161/cc.9.21.13671 (2010).

5 Reddi, A. H. *et al.* Initiation of Bone Development by Osteogenin and Promotion by Growth Factors. *Connective Tissue Research* **20**, 303-312, doi:10.3109/03008208909023901 (1989).

6 Kwon, H. J., Akimoto, H., Ohmiya, Y., Honma, K. & Yasuda, K. Gene expression profile of rabbit cartilage by expressed sequence tag analysis. *Gene* **424**, 147-152, doi:http://dx.doi.org/10.1016/j.gene.2008.07.036 (2008).

7 Lammi, M. J. *et al.* Hydrostatic pressure-induced changes in cellular protein synthesis. *Biorheology* **41**, 309-313 (2004).

8 Lomas, C., Tang, X. D., Chanalaris, A., Saklatvala, J. & Vincent, T. L. Cyclic mechanical load causes global translational arrest in articular chondrocytes: a process which is partially dependent upon PKR phosphorylation. *European cells & materials* **22**, 178-189 (2011).

9 De la Fuente, A. *et al.* Proteome Analysis During Chondrocyte Differentiation in a New Chondrogenesis Model Using Human Umbilical Cord Stroma Mesenchymal Stem Cells. *Molecular & Cellular Proteomics* **11**, doi:10.1074/mcp.M111.010496 (2012).

10 Kobayashi-Miura, M. *et al.* Rat Articular Cartilages Change Their Tissue and Protein Compositions During Perinatal Period. *Anatomia, Histologia, Embryologia* **45**, 9-18, doi:10.1111/ahe.12165 (2016).

11 Wang, Y. *et al.* Microarray analysis of proliferative and hypertrophic growth plate zones identifies differentiation markers and signal pathways. *Bone* **35**, 1273-1293, doi:http://dx.doi.org/10.1016/j.bone.2004.09.009 (2004).

12 Pogue, R. *et al.* A transcriptional profile of human fetal cartilage. *Matrix Biology* **23**, 299-307, doi:http://dx.doi.org/10.1016/j.matbio.2004.07.003 (2004).

13 Eames, B. F. *et al.* Mutations in fam20b and xylt1 Reveal That Cartilage Matrix Controls Timing of Endochondral Ossification by Inhibiting Chondrocyte Maturation. *PLOS Genetics* **7**, e1002246, doi:10.1371/journal.pgen.1002246 (2011).

14 Stelzer, C., Brimmer, A., Hermanns, P., Zabel, B. & Dietz, U. H. Expression profile of Papss2 (3′-phosphoadenosine 5′-phosphosulfate synthase 2) during cartilage formation and skeletal development in the mouse embryo. *Developmental Dynamics* **236**, 1313-1318, doi:10.1002/dvdy.21137 (2007).

15 Gaur, T. *et al.* Secreted frizzled related protein 1 regulates Wnt signaling for BMP2 induced chondrocyte differentiation. *Journal of Cellular Physiology* **208**, 87-96, doi:10.1002/jcp.20637 (2006).

16 Ohkawara, B. & Niehrs, C. P23. The role of Rspo3 in head cartilage morphogenesis. *Differentiation* **80, Supplement 1**, S24, doi:http://dx.doi.org/10.1016/j.diff.2010.09.029 (2010).

17 Stock, M., Schäfer, H., Fliegauf, M. & Otto, F. Identification of Novel Target Genes of the Bone-Specific Transcription Factor Runx2. *Journal of Bone and Mineral Research* **19**, 959-972, doi:10.1359/jbmr.2004.19.6.959 (2004).

18 Carpenter, D. *et al.* Characterization of two patched receptors for the vertebrate hedgehog protein family. *Proceedings of the National Academy of Sciences* **95**, 13630-13634, doi:10.1073/pnas.95.23.13630 (1998).
